# The State of Public Health Education and Science During and After the Fall of the Soviet Union: Achievements, Remaining Challenges, and Future Priorities

**DOI:** 10.3389/fpubh.2022.871108

**Published:** 2022-06-15

**Authors:** George Gotsadze, Nino Mirzikashvili, Dali Kekelidze, Sopio Kalandarishvili, Iagor Kalandadze, Ivane Abiatari, Akaki Zoidze

**Affiliations:** ^1^Curatio International Foundation, Tbilisi, Georgia; ^2^School of Natural Sciences and Medicine, Ilia State University, Tbilisi, Georgia; ^3^National Center for Disease Control and Public Health, Tbilisi, Georgia

**Keywords:** public health schools, public health education, public health research, public health science, USSR, former Soviet states

## Abstract

**Objectives:**

In the post–COVID-19 world, when the adequacy of public health workforce education is being critically re-evaluated, this study undertakes a historical analysis of how the educational and scientific field of public health developed during and after the fall of the Soviet Union in 1991. The study intends to historically contextualize public health education and science development in former Soviet Republics. It attempts to document achievements after gaining independence and identify remaining challenges that need to be addressed for advancing public health science and education in Former Soviet Union countries to better prepare them for future pandemics and address current health challenges of the nations.

**Methods:**

The study used a mixed-methods review approach combining both a literature review, information collection from the school's websites, and secondary analysis of the quantitative data available about scientific outputs—peer-reviewed articles.

**Results:**

During communist rule and after the fall of the Soviet Union, the main historical events seem to have shaped the public health field of former Soviet countries, which also determined its eventual evolution. The international efforts post-1991 were instrumental in shifting medically oriented conceptualization of public health toward Western approaches, albeit with variable progress. Also, while scientific output has been growing from 1996 to 2019, sub-regional differences remain prominent.

**Conclusion:**

The region seems to have matured enough that it might be time to start and facilitate regional cooperation of public health schools to advance the field of public health and research. Regional and country variabilities feature prominently in the volume and quality of scientific output and call for the immediate attention of national governments and international partners.

## Introduction

COVID-19 outbreak exposed challenges faced by nations' health systems when responding to health threats. The COVID-19 pandemic has made the public more aware of public health and the role its professionals play in addressing the pandemic (1). It revealed the importance of an adequately trained public health workforce required to take the lead in protecting citizens' health, expected to solicit and provide the evidence informing the design of the national responses; linking public health evidence with clinical services and offering targeted and scalable public health interventions in a way that protects and improves population's health at large. The COVID-19 pandemic demonstrated that even the states with relatively well-developed public health infrastructures faced problems during the COVID-19 outbreak. While the pandemic has revealed deficiencies in public health infrastructure and education of the public health workforce, it also created an opportunity to reassess and make substantial changes in the educational systems for public health for the long-term benefit (2, 3). From this perspective, COVID-19 is the biggest concern and momentum for reflection for the world, including for the Former Soviet Union (FSU) countries.

In the FSU countries, public health, as a field of science, is still evolving and much focused on educational activities and with little attention to research capacity building (4). In most countries, the inherited education system from the Soviet Union still medicalizes public health and conceptually limits epidemiology and public health to the study and control of communicable diseases (5). And the noted weaknesses translate into the lack of adequately trained public health workforce not having skills required for the 21st century. Therefore, even before the pandemic, FSU countries faced challenges in responding to their societies' communicable and non-communicable public health needs (6), which most likely constrained public health response to the COVID-19 pandemic in this part of the world (7, 8). Addressing populations' health needs requires many changes, most notably in the education system for the public health workforce. Furthermore, the pandemic has shown that there is a need to re-evaluate educational programs in many developing countries; early career researchers, policymakers in public health are all looking for ways to build public health workforce in the world of research, and this would require re-considering the skill-mix and competencies, creating trans-national inter-university teaching and learning environments (9)to better equip the students with the knowledge and skills enabling them to tackle future challenges and contribute to the global scientific conversation in the field of public health (10). Thus, pandemic provides an opportunity to consider fundamental changes and improve the approaches to, effectiveness in, and impact on public health education and the public health system to better cope with crises in the future (2, 11, 12).

To take stock, the study undertook a historical analysis of how the educational and scientific field of public health evolved in countries during and after the fall of the Soviet Union in 1991. The study intended to historically contextualize the developments, document achievements, and identify the challenges that need to be addressed by FSU countries for advancing public health science and education in this region to be better prepared for current and future health challenges.

## Methods

The study used a mixed-methods review approach combining information from a literature review combined with an analysis of data about scientific publications (13, 14) to reconstruct the developmental path of education and science in public health in the countries before and after the fall of the Soviet Union and contextualize these findings within the shared past of these countries while also looking at the scientific outputs and its evolution. Iterative literature reviews were used as we developed our understanding of the historical events and/or phases shaping the public health field during the Soviet times and after the fall of the Soviet Union. Findings from the literature review were contrasted with publicly available quantitative data about scientific outputs to arrive at conclusions.

In the first stage, we aimed at creating to the extent possible a complete list of the public health schools for post-soviet countries. We used Google search to find the websites of public health schools and construct the registry using the information available from the school's webpages. We limited the search to the 1992–2020 period and initially used the Russian and English search terms described below. Later, the search terms were translated into Azerbaijani, Armenian, Georgian, Turkmen, and Ukrainian. The search was repeated in these languages not to miss the schools that may not have English or Russian websites as they may have only served their nationals in the national language. After finding the school website, the information was extracted from web pages. The information not available in English or Russian was translated using Google translate. Our search terms included [School AND (“public health” OR “health management”)] AND (Armenia OR Azerbaijan OR Belarus OR Georgia OR Kazakhstan OR Kirgizia OR Kyrgyz Republic OR Tajikistan OR Uzbekistan OR Turkmenistan OR Ukraine OR Moldova OR Russia OR Russian Federation). We used structured information extraction to form the registry, which included the following data elements: school name, its location, country and city, the year of original establishment (i.e., if the school was established before 1991), the year of school name or profile change (where applicable) to see if the old schools changed the names and/or were converted into a new one, the type of programs offered in public health grouping into three broad categories (a) undergraduate, (b) graduate, or (b) postgraduate/doctoral, their membership/belonging to international societies. We attempted a granular comparison of the educational programs, which proved not possible due to a lack of comparable information in most instances. Therefore, this objective was dropped. In addition, we extracted qualitative historical information from the web pages, which also informed our literature review about the school and its historical evolution. A compiled list of schools for a given country was shared with colleagues in the country for validation (see Acknowledgments).

Next, the publicly available data sourced from SCOPUS (the largest abstract and citation database of peer-reviewed literature) (15) was used to measure research production for a given country. It helped evaluate the volume and quality of scientific output over the 1996–2019 period for each country and their regional sub-grouping. The 5-year time lag was allowed after the fall of the USSR for the new or re-formed schools to start producing research output relevant to the post-Soviet period. For measuring the volume of scientific output, we used six fields most relevant to public health from the SCOPUS database: “Health (Social Science),” “Health Information Management,” “Health Informatics,” “Health Policy,” “Health Professions,” and “Public Health.” For each country and year, we obtained a total number of published citable articles, total citations (excluding self-citations), and h-index. The data were enriched with the country's population for a given year obtained from the World Development Indicators (16) to obtain population-based comparable indicators for measuring scientific output measured with (a) the overall number of citable documents produced annually during 1996–2019 and their breakdown by countries, sub-regions, and 5-year periods to evaluate relative performance between the regions, countries, and over time, (b) a scientific field-specific h-Index for 1996-2019 measures both the productivity and citation impact of the articles relevant to public health science, and (c) a country-level h-Index for 1996–2019 to measure both the productivity and source impact of the country publications.

Finally, the study was carried out following relevant ethical guidelines of the author's institute for the research not involving human subjects.

## Context of Public Health Education and Science

### Origins and Development of Public Health: Early Days of the Soviet Union

The origins of social hygiene pre-dates the establishment of the Union of Soviet Socialist Republics (USSR). In 1917, the Russian government decreed the creation of the People's Healthcare Commissariat (the first governing body overseeing public health issues). It appointed Nikolai Semashko as the First People's Commissar of Health (14). He played a pivotal role in recognizing the role of socio-economic determinants of health and shaping the policies/decisions for public health interventions (17). Semashko's advanced views in public health were pioneering and have led to a new health care system focused on the universal treatment and prevention through sanitation, inspections, vaccinations, and attacks on “social” diseases such as tuberculosis, sexually transmitted disease, and alcoholism (18). Semashko also spearheaded education activities and, in 1922, established the first department of social hygiene at Moscow State University to serve the three medical schools in the city (17). After that, similar departments emerged in other cities of Russia and four Soviet republics: Azerbaijan, Belarus, Georgia, and Ukraine (see Table 1), where individuals with European education and/or professionally connected to Semashko championed the process (19). In other republics, such departments emerged later.

**Table 1:** Schools and departments in Former Soviet Union Countries.

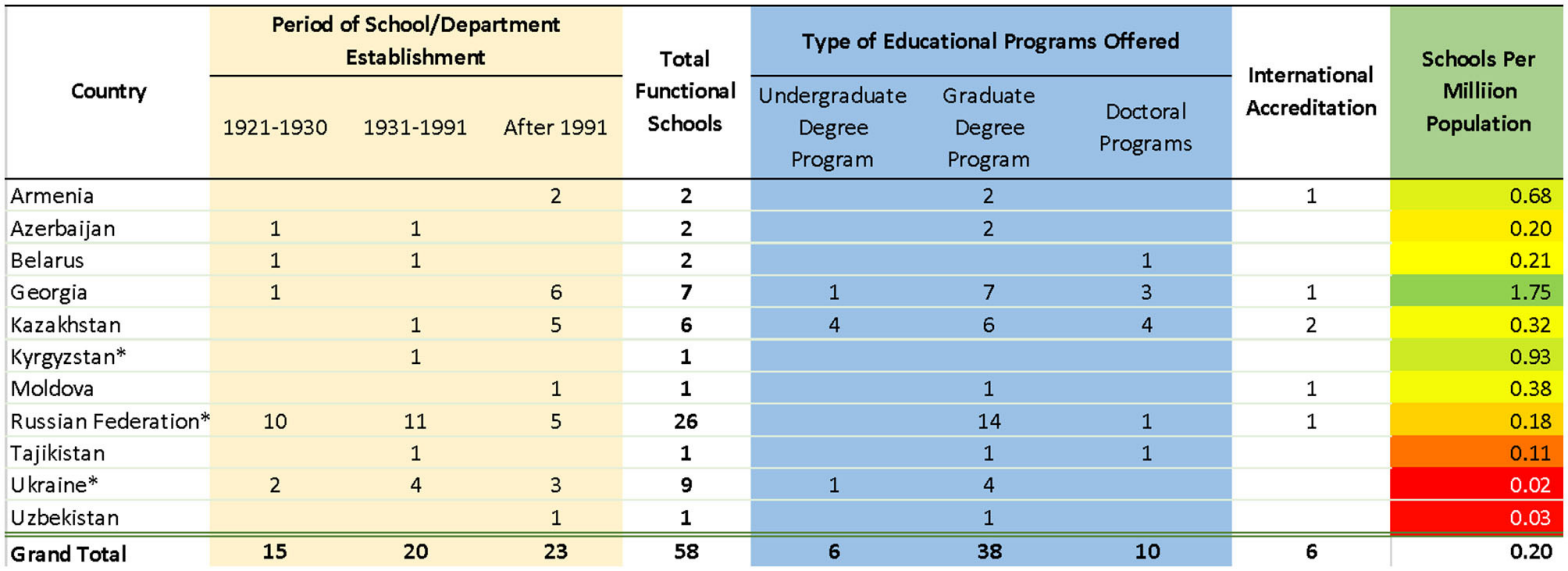

^*^Denotes incomplete information about the type of educational programs and degrees offered.

*The information is not available about Turkmenistan*.

Albeit, around 1930 the situation changed dramatically. The subject of social hygiene was considered non-Marxist, and the communist party suppressed social hygiene approaches (20).

### Establishment of the Soviet Public Health Concept 1930–1991

From 1941 until the mid-1960s, the scope of public health was further reduced and officially termed the “organization of health care.” The postwar period became quite difficult for science, as many outstanding specialists were repressed, and social hygiene was viewed as a Western, reformist, cosmopolitan, and bourgeois science (21), despite the evolution of the field carried on but with setbacks. During the 1950s and 1960s, the scope of *San-Epid* services was slightly broadened, and occupational and environmental health issues were added (6).

From 1931 to 1991, 20 schools/departments emerged throughout the Soviet Union, out of which 12 were opened before or during the war 1941–1945. However, the faculties suffered numerous transformations due to the USSR's modifying concept of public health (20). The last cohort of public health professionals trained by the Soviet system graduated in the 1990s. Even today, they continue to significantly influence public health development by inhibiting the implementation of modern public health approaches (22).

### Impact of Soviet Ruling on Public Health

Despite a good start, the development of the field became highly dependent on the political will of the communist party, which limited academic freedom, international professional contacts, and access to international literature, further aggravated by the fact that most professionals in the field lacked foreign language skills (23). The state allowed only a handful of research institutes and few libraries in Moscow to subscribe to international journals (24), which had detrimental effects on public health science and education development. As weak as the Soviet medical education system was, training individuals in health policy, research, and management were even worse (25).

Initially, the Soviet health system successfully scaled up essential interventions against infectious diseases and substantially improved the population's health relative to the starting point in the late 1920s (26). The *San-Epid* service protected health through communicable disease prevention and control, mass vaccinations and effective malaria surveillance, sanitary control of water supplies, hygienic waste disposal, and sewage (6). However, the Soviet public health theory and practice failed to address the unfolding epidemic of non-communicable diseases and relevant risk factors. They missed certain “social” diseases, including abuse of psychoactive substances (tobacco, alcohol, and drugs) and the newly emerged, for example, HIV/AIDS (6, 20). Consequently, the USSR failed to prevent the decline of many health indicators experienced after 1964, which can be explained by sacrificing social to military concerns during the cold war (6) and inadequately preparing the public health workforce to deal with growing health threats.

### Details of Post-1991 Developments

After the disintegration of the Soviet Union in 1991, public health structures and educational systems were largely destroyed, and infectious diseases such as diphtheria, sexually transmitted infections, and tuberculosis re-emerged (6). While the Western concept of public health became popular, the transformation of the *San-Epid* system into the new public health system proved challenging with limited funding, shortages of adequately trained staff, and little exposure to modern concepts of public health (6, 22). One of the main problems was moving from a narrow/medicalized conceptualization of public health to one addressing the broader health determinants (27). Clear definitions were lacking for public health, public health workforce, public health skills, and competencies. There was a need for a mind shift followed by faculty development in specific subjects and teaching/research methodology (28).

Schools of “new” public health began to appear in the early 1990s, and the World Bank, the European Union, and other donors helped establish these schools (29). Several medical schools reformed their sanitary-hygienic faculties into public health faculties offering reduced clinical and expanded epidemiology training, and schools modeled on European or North American approaches emerged (5). The international collaboration helped develop MPH and doctoral courses with new curriculums (28). Some newly established schools became members of international associations, such as *the Association of Schools of Public Health in the European Region* (ASPHER) (30). Finally, these schools were supported to focus on each component of the triad of education, research, and service to the community.

Due to international collaboration since 1991, 23 (40%) public health schools/departments emerged (see Table 1), out of which 16 emerged since 2000. Schools developed more graduate and doctoral programs but paid little attention to undergraduate education. Georgia, Kyrgyzstan, and Armenia have a higher supply of schools per population, while Ukraine and Uzbekistan are lagging (see Table 1). While all schools went through a national accreditation process (where required), the quality standards/ requirements imposed by the national accreditation system raise questions. Only six schools sought international accreditation, most likely reflecting adherence to their educational programs and curriculum to Western standards. While some countries are taking progressive steps to develop Western-type public health education and science, the progress has been slow and uneven as some schools (e.g., Azerbaijan, Belarus, Kyrgyzstan, and Tajikistan) still retain a medical orientation of public health. In contrast, others employ Western workforce education approaches that include research capacity development.

**Figure 1:**
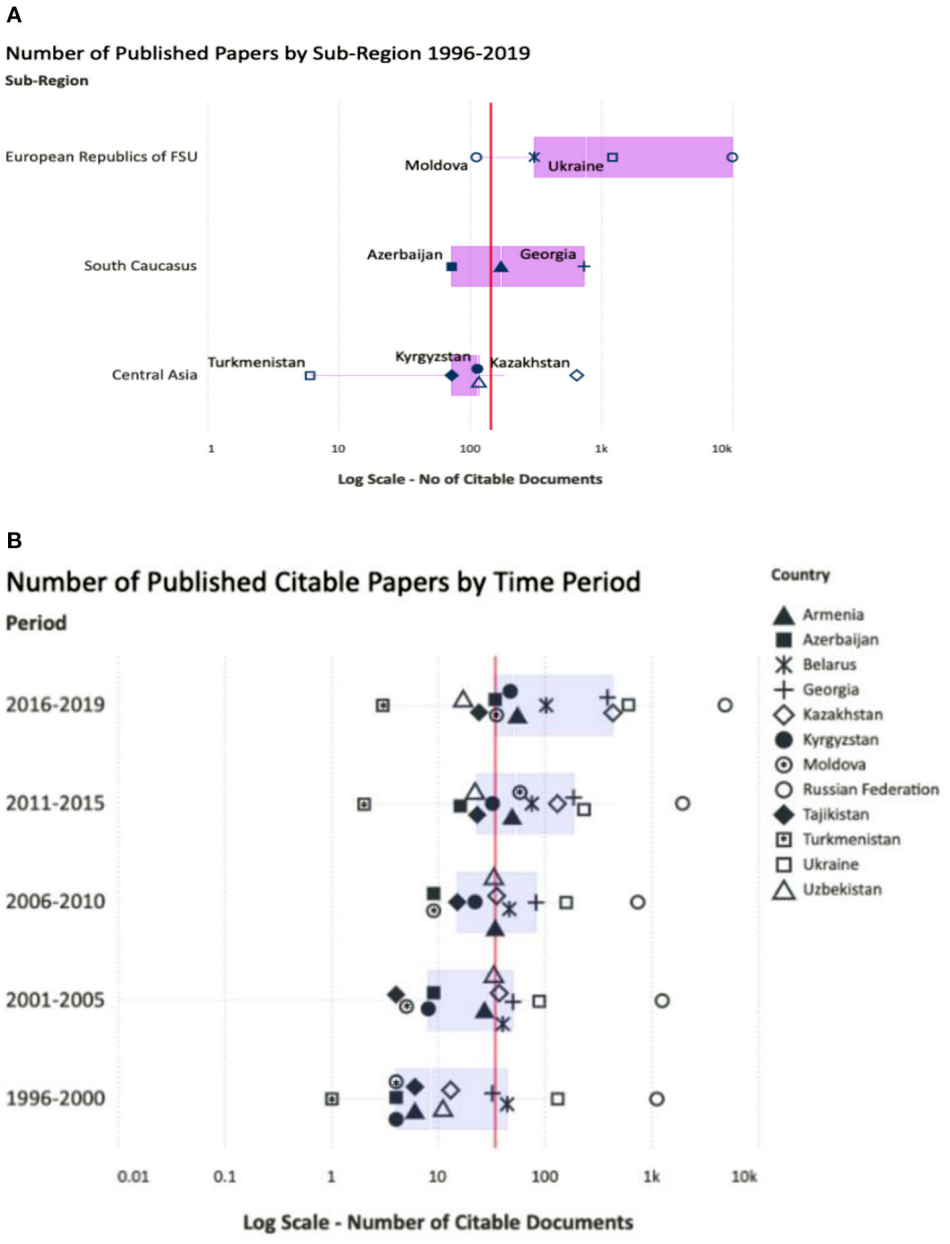
**(A)** Published citable articles in Web of Science database during 1996–2019 by sub-regions and **(B)** number of published citable articles by the time about here.

**Figure 2:**
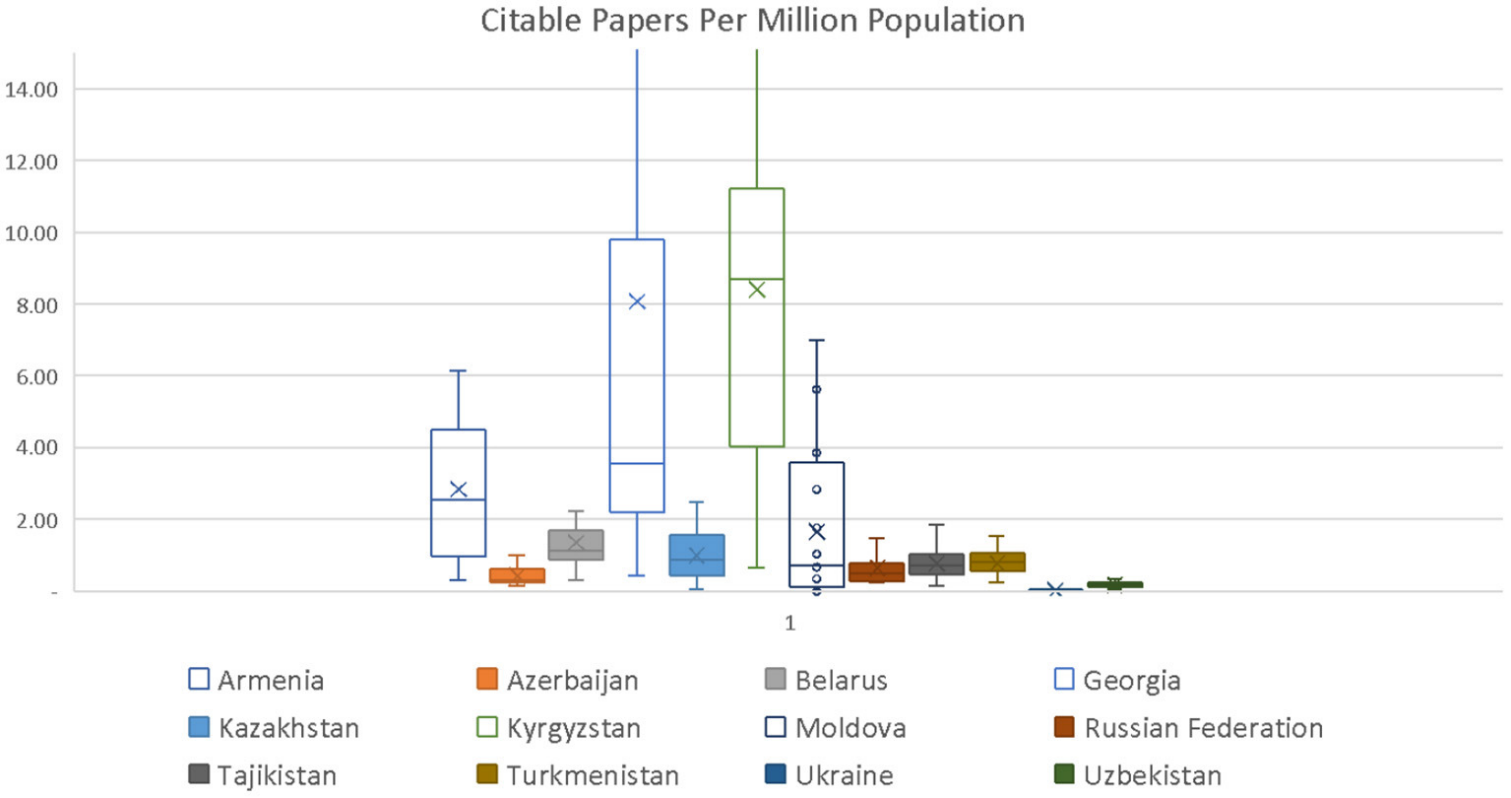
Published citable articles in Web of Science database during 1996–2019 adjusted to population size.

Overall, scientific output grew from 1996 to 2019 with significant sub-regional and country variations (see Figure 1A). Published articles increased in all countries, but Georgia and Kazakhstan posted the fastest growth (see Figure 1B). Russian Federation and Ukraine lead with a total volume of published articles, but Kyrgyzstan, Georgia and Armenia, and Moldova (in the growing order) have the highest per-capita scientific production (see Figure 2). And Armenia, Georgia, and Kyrgyzstan publish better quality articles when compared to other post-Soviet countries (31).

**Figure 3:**
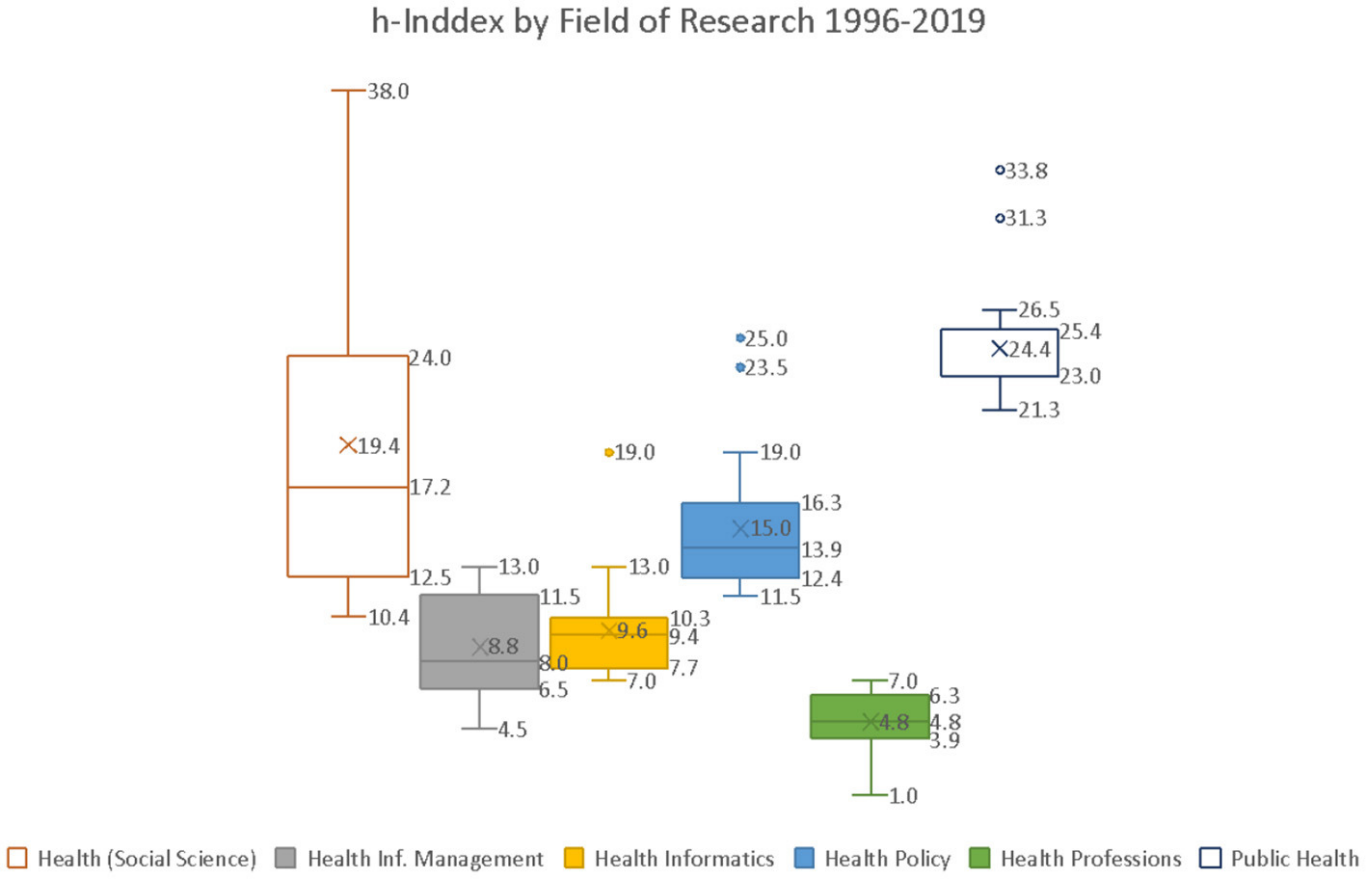
h-Index by the field of research 1996–2019.

Predominant articles are in the public health field, followed by health informatics and information management. While health and social science and health policy are less of a focus, most impactful articles come in “health social science” (median = 17.2) and “health policy” (median = 13.9) along with “public health” (median = 23.0) (see Figure 3). The Russian Federation (h = 28) and Ukraine (h = 19), followed by Georgia (h = 18) and Kyrgyzstan (h = 12), are leading in their respective sub-regions and seem to be producing higher-quality scientific outputs.

## Discussion

The findings lead to several conclusions, which must be considered with the study limitations. While efforts were made to identify all schools in FSU countries offering public health education, we may have missed some schools, but not many, as we tried to validate our school registry for a country with the help of our peers in a given country. Therefore, the number of schools may not be a complete census, but they indeed reveal trends and are accurate in illuminating the developmental path. The inventory of educational programs arises from program descriptions on the school's website. Some were found incomplete or not comprehensive, possibly indicating weaknesses in academic/accreditation standards of a country or insufficient investment in program presentation on the Web. Therefore, our attempt to look closely at the teaching programs and competencies offered to students proved not feasible and in that imposed limitations on the findings. The data about published articles is not without constraints because the database has problems covering articles in languages other than English (32). While absolute measures in the article could underestimate the quantity and impact of research output from post-Soviet countries, we believe the overall trend is reflective of the developmental path.

Notwithstanding these limitations, we conclude: First, public health education and science during Soviet times have been subjected to ideological swings throughout history, most likely also predetermining and negatively influencing the post-1991 developmental path. Second, while significant health gains were attained during the early days of the Soviet Union, public health and its workforce proved not sufficiently prepared to face emerging challenges and mount adequate response during the later years of the USSR. Third, international efforts post-1991 were instrumental in shifting medically oriented conceptualization of public health toward Western approaches, *albeit* with variable and, to a degree slow progress. And fourth, the legacy of the USSR is still present in the region, possibly due to a sizable cadre of individuals who graduated in the 1990s and who prevent renewed conceptualization of public health and are setting the standards of today's public health education? Fifth, the quality standards and requirements of the national accreditation, especially in countries with a small number of schools, could be a factor supporting this legacy and delaying the needed modifications for education and workforce development, capable of meeting the current health needs of their nations. Finally, while the Soviet legacy looks durable, some schools have taken progressive steps, developed new programs, and sought international accreditation. The broadening focus of education and embracing research seems to be rendering initial results with a growing number of scientific outputs in the field, albeit with the remaining need to enhance quality with more impactful articles. Regional and country variabilities feature prominently and call for the immediate attention of national governments and international partners.

All these findings lead to a thought that due to Western support in the past, the region seems to have emerged enough that it might be time to jump-start regional cooperation of public health schools creating an environment where the success of some countries could be shared, and experiences exchanged. Instead of relying on nationally developed accreditation standards, regionally developed accreditation standards for educational programs could be elaborated and proposed for national use. A regional accreditation body could be established or better linked to ASPHER, and the quality of programs could be further improved. Regionally relevant collaborative research could be facilitated by establishing regional funding bodies/channels for research.

It seems the regional office of the World Health Organization and the development banks need to work together and with important regional stakeholders to facilitate collaboration and accelerate shifts in public health education and science so much needed for the region to advance the skills and capabilities of its public health workforce. Such actions, when successful, could bring greater resilience to national health systems and better prepare them for the next health emergencies.

### Practical Implications and Key Takeaways

While some public health schools have taken progressive steps, developed advanced programs, and sought international accreditation from international societies, most schools in the FSU countries are lagging on the quality of the educational programs. They lack the modern approaches necessary to produce a well-qualified public health workforce. Thus, greater regional collaboration between the schools to improve curriculums could accelerate needed transformations and help improve the quality and relevance of the educational programs to the current demands placed on the public health workforce. And eventually, if such cooperation is reinforced with the regional accreditation body and/or regionally developed academic standards, the sustainability of regional cooperation could be even further enhanced.

Regional and country variabilities feature prominently in the volume and quality of scientific output and call for immediate attention. Collaborative research funding facilitating cross-border researcher partnership could be a promising mechanism for further production of region-relevant and more impactful research.

However, such collaborations to emerge would require first the recognition of the need and, thereafter, the attention of national governments to these issues. And it seems that through active engagement from the regional office of the World Health Organization for Europe and financial support from the development banks and partners, shifts in public health education and science in this region could be accelerated, and the skills and capabilities of its public health workforce could be advanced.

## Data Availability Statement

The original contributions presented in the study are included in the article/supplementary material, further inquiries can be directed to the corresponding author.

## Author Contributions

Study conception and design: GG. Acquisition of data: NM, DK, and SK. Data analysis, interpretation, and drafting of the manuscript: GG and NM. Conclusions and critical revisions: IK, IA, and AZ. All authors read and approved the final manuscript.

## Funding

Ilia State University and Curatio International Foundation funded the research in the year 2021.

## Conflict of Interest

The authors declare that the research was conducted in the absence of any commercial or financial relationships that could be construed as a potential conflict of interest.

## Publisher's Note

All claims expressed in this article are solely those of the authors and do not necessarily represent those of their affiliated organizations, or those of the publisher, the editors and the reviewers. Any product that may be evaluated in this article, or claim that may be made by its manufacturer, is not guaranteed or endorsed by the publisher.
